# P033. Headache and commuting: preliminary data in a group of workers

**DOI:** 10.1186/1129-2377-16-S1-A79

**Published:** 2015-09-28

**Authors:** Ennio Pucci, Giuseppe Taino, Marcello Imbriani, Alfredo Costa, Silvano Cristina, Fabio Antonaci

**Affiliations:** Headache Science Center, University Consortium for the Study of Adaptive Disorders and Headache (UCADH), Department of Brain and Behavioral Sciences, University of Pavia, IRCCS “C. Mondino”, Pavia, Italy; U.O. Hospital Occupational Medicine, Foundation IRCCS “S. Maugeri”, Pavia, Italy; Department of Public Health, Experimental and Forensic Medicine, University of Pavia, Pavia, Italy

## Introduction

The different forms of primary headache disorders are highly prevalent which, taken together, affect a greater extent of people of working age. Risk factors of headache triggers can be physical, psycho-social or organizational (work in shifts, night work, working conditions non-ergonomic, etc.). Commuting is a phenomenon which consists in the double daily shift of people moving, usually by public transport.

## Objective

The aim of this study was to study the phenomenon of commuting, especially the prevalence, in a group of workers of a chemical industry.

## Patients and methods

Health surveillance and medical history questionnaires focused on employment and on the presence of primary headache in 95 workers of a chemical industry (91 M, 4 F). Night shift work interested 52.6% of workers, while 47.4% worked during the day. The diagnosis of migraine was defined according to the criteria of the ICHD-III beta version.

## Results

The analysis of the questionnaires and the processing of the results showed that the form of primary headache with higher prevalence, in both night shift workers and day workers, was represented by migraine without aura (51.5% of all headache workers) followed by tension-type headache (42.5%) and by migraine with aura (6%). The different prevalence of primary headaches in the two groups of workers (shift and day workers) did not reach statistical significance. We then decided to consider instead the commuting variable since 46% of night shift workers were also commuters. Processing of the data of the subgroup showed a statistically significant association between the prevalence of primary headache and commuting/night shift (p < 0.05).

## Conclusions

The commuters/shift workers are more prone to develop a primary headache, especially migraine without aura. Further investigations are needed to better clarify the association between primary headache and commuting/shift working, and also, more generally, “unusual” rhythm of work.

Written informed consent to publication was obtained from the patient(s).

